# Safety and Effectiveness of Transvenous Lead Extraction in Patients with Infected Cardiac Resynchronization Therapy Devices; Is It More Risky than Extraction of Other Systems?

**DOI:** 10.3390/ijerph19105803

**Published:** 2022-05-10

**Authors:** Paweł Stefańczyk, Dorota Nowosielecka, Anna Polewczyk, Łukasz Tułecki, Konrad Tomków, Wojciech Jacheć, Ewa Lewicka, Andrzej Tomaszewski, Andrzej Kutarski

**Affiliations:** 1 Department of Cardiology, The Pope John Paul II Province Hospital of Zamość Poland, 22-400 Zamość, Poland; paolost@interia.pl (P.S.); dornowos@wp.pl (D.N.); 2 Department of Physiology, Patophysiology and Clinical Immunology, Collegium Medicum of Jan Kochanowski University, 25-369 Kielce, Poland; 3 Department of Cardiac Surgery, Świętokrzyskie Cardiology Center, 25-736 Kielce, Poland; 4 Department of Cardiac Surgery, The Pope John Paul II Province Hospital of Zamość Poland, 22-400 Zamość, Poland; luke27@poczta.onet.pl (Ł.T.); konradtomkow@wp.pl (K.T.); 5 Department of Cardiology, Faculty of Medical Science in Zabrze, Medical University of Silesia, 40-752 Katowice, Poland; wjachec@interia.pl; 6 Department of Cardiology and Electrocardiotherapy, Medical University of Gdańsk, 80-416 Gdańsk, Poland; ewa.lewicka@gumed.edu.pl; 7 Department of Cardiology, Medical University of Lublin, 20-059 Lublin, Poland; benecho2008@gmail.com (A.T.); a_kutarski@yahoo.com (A.K.)

**Keywords:** transvenous lead extraction, cardiac resynchronization therapy, infectious indications, safety and effectiveness

## Abstract

Background: Transvenous lead extraction (TLE) in patients with implantable cardioverter defibrillator (ICD) and cardiac resynchronization therapy (CRT) devices is considered as more risky. The aim of this study was to assess the safety and effectiveness of TLE in patients with infected CRT systems. Methods: Data of 3468 patients undergoing TLE in a single high-volume center in years 2006–2021 were analyzed. The clinical and procedural parameters as well as the efficacy and safety of TLE were compared between patients with infected CRT and pacemakers (PM) and ICD systems. Results: Infectious indications for TLE occurred in 1138 patients, including 150 infected CRT (112 CRT-D and 38 CRT-P). The general health condition of CRT patients was worse with higher Charlson’s comorbidity index. The number of extracted leads was higher in the CRT group, but implant duration was significantly longer in the PM than in the ICD and CRT groups (98.93 vs. 55.26 vs. 55.43 months *p* < 0.01). The procedure was longer in duration, more difficult, and more complex in patients with pacemakers than in those in the CRT group. The occurrence of major complications and clinical and procedural success as well as procedure-related death did not show any relationship to the type of CIED device. Mortality at more than one-year follow-up after TLE was significantly higher among patients with CRT devices (22.7% vs. 8.7%) than among those in the PM group. Conclusion: Despite the greater burden of lead and comorbidities, the complexity and efficiency of removing infected CRT systems is no more dangerous than removing other infected systems. The duration of the implant seems to play a dominant role.

## 1. Introduction

In recent years, the number of patients with cardiac implantable electronic devices (CIED) has increased significantly. A special group are patients with dyssynchronous heart failure, in whom the use of cardiac resynchronization therapy (CRT) such as CRT-defibrillators (CRT-D) and pacemakers (CRT-P) improves symptoms and reduces mortality, as confirmed in many randomized clinical trials [1,2,3]. With the increase in the number of cardiac implantable electronic devices in patients with heart failure, a significant increase in the number of infections associated with CIEDs has been observed. This is probably due to the presence of comorbidities such as diabetes mellitus, chronic renal failure and more frequent replacement procedures [4]. Olsen et al. reported that the incidence of device-related infections over the lifetime of the device was 2.18% (1.78–2.64) for cardiac resynchronization therapy (CRT)-pacemakers, and 3.35% (2.92–3.83) for CRT-defibrillators [5]. Transvenous lead extraction (TLE) is an integral part of the lead management strategy and the gold standard for treatment of CIED infections and lead failure [6,7,8,9,10,11]. The effectiveness of TLE is high (more than 90% in general) but rates of major complications vary between studies from 0.4 to 3.4%, whereas mortality risk is 0.00–1.86% [8,9,10,11,12,13].

Transvenous lead extraction of permanently implanted coronary sinus (CS) leads and ICD leads is widely believed to present greater risks than the removal of other leads [12,13,14,15,16,17]. The increased difficulty in removing the left ventricular lead is explained by the thin wall of the coronary sinus and the smaller diameter of the electrode body, but there are limited data to support this hypothesis. Most studies of lead extraction provide information on leads from the right atrium and right ventricle, but only a few studies investigated the extraction of CS leads [18,19,20,21,22,23]. In this study, we analyzed our experience with cardiac resynchronization lead extraction due to infection from the perspectives of efficacy, safety, and complication rate.

## 2. Materials and Methods

### 2.1. Study Population

All transvenous lead extraction procedures performed between March 2006 and July 2021 at a single high-volume center were screened. Patient and lead data were retrospectively analyzed from a computerized database. All participants provided written informed consent prior to study enrollment, and before the TLE procedure as medically indicated. Multiple parameters including patient demographics, comorbidities, procedural success, device type, major complications, and mortality were incorporated into the database prospectively. In patients with non-infective TLE indications, antibiotic prophylaxis was based on a bolus of a first-generation cephalosporin administered 1 h before TLE. In patients with an infective indication for TLE, the antibiotic regimen was culture-guided. In these patients, a targeted antibiotic regimen was then continued for at least 2 weeks in the case of pocket infection and for more than 4 weeks in the case of endocarditis or systemic bacteriaemia. Reimplantation was performed once targeted antibiotic therapy was effective and blood cultures after TLE were negative [9,10,11].

The study groups were formed on the basis of the different types of infected devices extracted: all pacemakers (*n* = 756), all ICD (*n* = 232), all CRT systems (*n* = 150) including CRT-P (*n* = 38) and CRT-D (*n* = 112). The results for the different groups were analyzed and compared.

### 2.2. Lead Extraction Procedure

Indications for TLE, procedure effectiveness, and complications were assessed according to the 2009 and 2017 HRS consensus and 2018 EHRA guidelines [8,9,10,11]. The efficacy of TLE was determined based on the percentage of procedural success and clinical success including complete and partial radiographic success. Procedural success was defined as the removal of all targeted leads and lead material from the vascular space with the absence of any permanently disabling complication or procedure-related death. Clinical success was defined as the removal of all targeted leads or retention of a small portion (<4 cm) of the lead that did not negatively impact the outcome goals of the procedure (i.e., residual lead did not increase the risk of perforation, embolic events, perpetuation of infection, or cause any undesired outcome), absence of any permanently disabling complication or procedure-related death [8,9,10,11].

The complications of TLE were also defined as major complications such as those that were life threatening, resulted in significant or permanent disability or death, or required surgical intervention [8,9,10].

A CRT TLE (CRT group) was defined as a TLE in a patient with a CRT system incorporating a CS lead, including both CRT defibrillators (CRT-D) and pacemakers (CRT-P). A non-CRT TLE (non-CRT group) was defined as all other system and lead extractions in patients without a CRT system.

In most procedures, standard stylets were used to stiffen the leads. Locking stylets (Liberator Locking Stylet, Cook Medical Inc., Bloomington, IN, USA) were used only for extraction of the oldest leads when estimated risk of lead fracture was high. Simple traction or traction on a locking stylet with insulation-bound suture was very rarely applied (usually in patients with infection, when prolonged temporary pacing was not planned). Lead extraction was performed using mainly non-powered mechanical telescoping polypropylene sheaths (Byrd Dilator Sheaths, Cook Medical Inc., Bloomington, IN, USA) of all diameters and lengths, and using various stylets. When the polypropylene telescoping sheaths appeared ineffective, powered mechanical sheath systems (Evolution Mechanical Dilator Sheath, Cook Medical Inc., USA; TightRail Rotating Dilator Sheath, Spectranetics, Colorado Springs, Co, USA) were used. A combined approach, using two or more different (jugular, subclavian, femoral) access sites, was selected when conventional methods were insufficient. Laser and electrosurgical dissection sheaths were not used.

All extraction procedures were performed following different organizational models spanning 15 years of experience. At the beginning of lead extraction, the procedures were performed in the electrophysiology laboratory using intravenous analgesia/sedation [24]; then, the recommended safety precautions were observed to perform more complex and risky procedures in the operating theater, and finally in the hybrid room under general anesthesia. Over the past 6 years, the core extraction team has consisted of the same highly experienced TLE operator, experienced echocardiographer and dedicated cardiac surgeon [25,26,27].

### 2.3. Dataset and Statistical Methods

Statistical analyses were carried out using Statistica v. 13.3 (TIBCO Software Inc., Palo Alto, CA, USA). Categorical variables were expressed as counts and percentages, and continuous variables as either the mean and standard deviation (SD) or median. The variables were compared using the nonparametric Chi2 test with Yates correction (dichotomous data) or the unpaired Mann–Whitney *U* test (continuous data), as appropriate. A *p*-value of less than 0.05 was considered statistically significant.

### 2.4. Approval of the Bioethics Committee

All patients gave their informed written consent to undergo TLE and use anonymous data from their medical records, approved by the Bioethics Committee at the Regional Chamber of Physicians in Lublin no. 288/2018/KB/VII. The study was carried out in accordance with the ethical standards of the 1964 Declaration of Helsinki.

## 3. Results

A total of 3546 patients underwent lead extraction procedures (61%male), age 5–94 (66.7 ± 14.96). Indications for TLE included: systemic infection in 22.4% of patients, local isolated pocket infection in 9.6%, and non-infective indications in 67.9% of patients. Among patients with infection, 150 were patients with CRT, representing 4% of all patients with TLE and 13% of all patients with infection. The mean dwell time of the oldest infective lead in one patient was 91.58 ± 69.23 months; the time from last CIED procedure in one patient was 35.18 ± 32.15 months.

**Figure 1:**
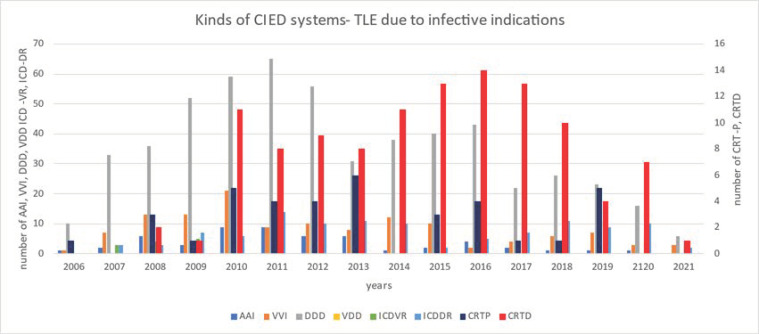
Annual number of transvenous lead extraction procedures procedures, taking into account the type of devices.

The annual number of TLEs due to infectious reasons varied from year to year. Most CRT systems were removed in 2014–2018 (Figure 1).

For the purposes of analysis, the study population with infective CIED was divided into five groups: 1—all pacemakers (AAI, VVI, DDD, VDD), 756 patients, 2—ICDs all (VVI, DDD), 232 patients, 3—CRT-P, 38 patients, 4—CRT-D, 112 patients, and 5—all CRT systems, 150 patients. Tables summarize the indications for the initial implantation of devices and present the specific patient-, system- and procedure-related risk factors as well as analyze complexity, efficacy, complications of the procedures and long-term mortality after TLE.

Table 1 presents detailed indications for the implantation of particular types of devices in the study population.

Analysis of the clinical factors showed that CRT-group patients were slightly younger, there were more male patients, with worse functional NYHA class, decreased LVEF, more frequent renal failure, diabetes mellitus and finally, higher Charlson’s comorbidity index. However, the type of infection (infective endocarditis or pocket infection) did not show any relationship to the type of CIED system (Table 2).

The number of leads in the heart before TLE, presence of ≥ 4 leads in the heart and number of procedures before lead extraction were more frequent in the CRT system groups. Similarly, the number of extracted leads in one patient and extraction of three or more leads were more frequent in CRT groups. Implant duration expressed as the oldest extracted lead dwell time in patient, average extracted lead dwell time in patient, average lead duration in analyzed group, and cumulative dwell time of extracted lead in the patient was significantly longer in PM (AAI, VVI, DDD, VDD) than in the CRT and ICD groups. In the group of CRT patients, the highest percentage of passive fixation leads was found. The risk of infectious complications according to the PADIT [28] scale was highest in patients with CRT-D. Estimated risk of major complication using SAFeTY-TLE calculator [29] (expressed in points and as probability percentage) was lower in the CRT and ICD groups. Multiple leads to be removed seemed to be a less important risk factor than implant duration (Table 3.)

A comparison of the TLE complexity of different CIED systems showed that the duration of the procedure was longer in the "all pacemakers" group than in the CRT group. Additionally, the appearance of most technical difficulties (problems) was less frequent in the CRT group than among all pacemaker carriers. Differences did not reach statistical significance, but the direction of the trend was visible. (Table 4).

The occurrence of any major complications, the need for rescue cardiac surgery, damage to the tricuspid valve during TLE, the complete clinical success and complete procedural success, and deaths related to the procedure (intra-, postoperative) did not show any relationship with the type of CIED removed (Table 5).

**Figure 2:**
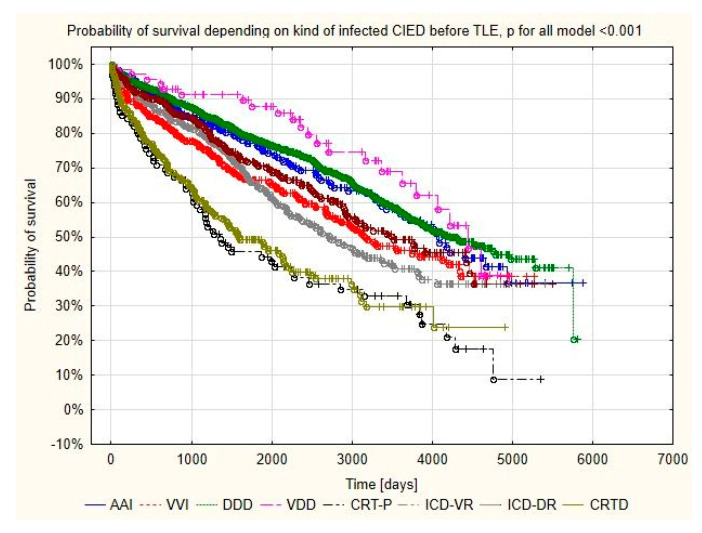
Kaplan–Meier survival curves of patients with the different types of infected CIED.

The prognosis analysis after TLE for infectious reasons showed that the percentage of deaths in the CRT group was higher than that in the pacemaker group (64% vs. 45.9% *p* < 0.001), but no association was shown for the 48 h and 1 month mortality with the type of device removed. However, mortality in more than 1 year of follow-up after TLE was significantly higher in patients with CRT (22.7%) than in the group with pacemakers (8.7%) *p* < 0.001, as was the mortality at 3 years after TLE (30.7%) vs. (10.8%) *p* < 0.001 (Table 6, Figure 2).

## 4. Discussion

Transvenous lead extraction of permanently implanted coronary sinus (CS) leads and ICD leads is widely believed to present greater risks than the removal of other leads [12,13,14,15,16,17]. The greater difficulty in removing the left ventricular lead is explained by the thin wall of the coronary sinus and the smaller diameter of the electrode body, but there are limited data to support this hypothesis. Most reports provide information on TLE for leads from the right atrium and right ventricle, but only a few relate to the extraction of CS electrodes [19,20,21,22,23]. The present study showed that despite worse general health condition, higher lead burden and number of extracted leads in the CRT group, the complexity of the procedure, complication rate, the effectiveness of TLE, and the mortality associated with the procedure were not worse than in the PM and ICD groups. However, it should be emphasized that the lead dwell time in the CRT group was significantly shorter compared to that in the PM and ICD groups. Thus, multiple leads appear to be a less significant risk factor than the implant duration. Additionally, the old models of the double-coil ICD lead represented an accepted risk factor for serious complications of TLE [12,13,14,15,16,17], but the latest models do not generate additional risk [30,31,32], similar to the extraction of modern CS leads [23,30]. A separate, important problem is sudden temporary loss of cardiac resynchronization, which may lead to severe circulatory deterioration in good CRT responders [33,34,35], but this phenomenon is not considered a complication of TLE.

The current study also found that simple, cheap and conventional tools (non-powered polypropylene mechanical sheaths) used as first-line support help to achieve excellent results in CRT patients. The procedure-related major complications for all infected patients was 2.6%, and was higher than reported in the ELECTRa (1.7%) [36] and 5000 lead extracted Cleveland Clinic Series (1.8%) [17] and in the study by Gould et al. (1.4%) [37]; however, no major complication- and procedure-related deaths (0%) were found in the CRT group. The all-cause 30-day mortality rate was 2.7% with no statistically significant difference between the two groups (CRT 4.6%, n = 7 vs. non-CRT 2.4%, n = 24, *p* = 0.247). These results confirmed that the serious complications associated with the TLE procedure and the mortality rate in patients with CRT are not higher compared to those in the PM / ICD groups, despite the greater number of comorbidities and the greater number of leads removed in each case. The specificity of postoperative management involving the extraction of an infected CRT system during antibiotic therapy is associated with frequent deterioration of the hemodynamic status of patients [33,34,35] and more difficult, more complicated reimplantation of CRT [38].

## 5. Conclusions

1. In spite of the higher lead and co-morbidity burdens, TLE of infected CRT systems is no more dangerous or difficult than removing infected pacemaker and ICD systems. The main factor influencing the effectiveness of the procedure remains implant duration.
2. Long-term survival after removal of infected CRT systems is worse than that after removal of other systems, but short term mortality is comparable with that of non-CRT patients. It is related to the worse clinical presentation of CRT patients at baseline.

## Figures and Tables

**Table 1:** Indications for initial implantation of CIED.

	Number of Patients	SSS	II–III Degree of a-v Block	HF	HF with LBBB
AAI	54	54	0	0	0
VVI	129	29	100	0	0
DDD	556	236	320	0	0
VDD	17	0	17	0	0
CRT-P	38	0	6	0	32
ICDVR	122	0	20	102	0
ICDDR	110	0	0	110	0
CRT-D	112	0	0	12	100

HF—heart failure, LBBB—left bundle branch block, SSS—sick sinus syndrome.

**Table 2:** Clinical characteristics of patients with different types of CIED system.

	Pacemakers All (AAI, VVI, DDD, VDD)	ICDs All (VVI, DDD)	CRT-P	CRT-D	All CRT Systems (CRT-P Plus CRT-D)
	Group 1 N = 756	Group 2 N = 232	Group 3 N = 38	Group 4 N = 112	Group 5 N = 150
	Mean ± sd N (%)	Mean ± sd N (%)	Mean ± sd N (%)	Mean ± sd N (%)	Mean ± sd N (%)
Chi^2^ test, “U” Mann–Whitney test		1 vs. 2	1 vs. 3	1 vs. 4 2 vs. 4	1 vs. 5 2 vs. 5
Patient’s age during TLE	69.96 ± 14.17	64.26 ± 12.63 *p* < 0.001	70.26 ± 9.57 *p* = 0.488	67.42 ± 10.23 *p* < 0.001 *p* = 0.039	68.14 ± 10.11 *p* = 0.002 *p* = 0.004
Patient’s age during first system implantation	61.10 ± 15.82	59.62 ± 12.87 *p* = 0.009	62.92 ± 9.966 *p* = 0.986	62.42 ± 10.55 *p* = 0.821 *p* = 0.049	62.55 ± 10.37 *p* = 0.855 *p* = 0.021
Sex (% of female patients)	272 (35.98)	32 (13.79) *p* < 0.001	10 (26.32) *p* = 0.298	18 (16.07) *p* < 0.001 *p* = 0.690	28 (18.67) *p* < 0.001 *p* = 0.257
Etiology other than IHD	403 (53.31)	152 (65.51) *p* < 0.001	18 (47.37) *p* = 0.583	47 (41.96) *p* = 0.032 *p* < 0.001	65 (43.33) *p* = 0.032 *p* < 0.001
NYHA III or IV class	64 (8.466)	65 (28.02) *p* < 0.001	15 (39.47) *p* < 0.001	50 (44.64) *p* < 0.001 *p* = 0.002	65 (43.33) *p* < 0.001 *p* = 0.003
LVEF (%)	54.69 (10.25)	37.19 (14.19) *p* < 0.001	36.21 (13.75) *p* < 0.001	30.77 (12.37) *p* < 0.001 *p* < 0.001	32.15 (12.91) *p* < 0.001 *p* < 0.001
Renal failure moderate(creatinine >1.3—≤2.2 mg%)	136 (17.10)	61 (26.29) *p* = 0.008	9 (23.68) *p* = 0.502	29 (25.89) *p* = 0.063 *p* = 0.959	38 (25.33) *p* = 0.049 *p* = 0.929
Renal failure severe or hemodialysis(creatinine ≥ 2.3 mg%)	42 (5.556)	22 (9.48) *p* = 0.049	4 (10.54) *p* = 0.356	10 (8.93) *p* = 0.234 *p* = 0.974	14 (9.33) *p* = 0.117 *p* = 0.896
Renal failure (all),creatinine ≥ 1.3 mg%	178 (22.66)	83 (35.77) *p* = 0.003	13 (34.22) *p* = 0.191	39 (34.82) *p* = 0.014 *p* = 0.958	52 (34.66) *p* = 0.006 *p* = 0.911
Diabetes	158 (20.90)	68 (29.31) *p* = 0.010	13 (34.22) *p* = 0.081	35 (31.25) *p* = 0.020 *p* = 0.808	48 (32.00) *p* = 0.004 *p* = 0.657
Carlson’s index (points)	4.86 ± 3.559	5.78±4.01 *p* = 0.002	6.40 ± 4.175 *p* = 0.022	6.01 ± 3.85 *p* = 0.003 *p* = 0.624	6.11 ± 3.92 *p* < 0.001 *p* = 0.430
TLE indications—more exact division of infective indications					
Lead related infective endocarditis certain (with pocket infection or without)	375 (49. 60)	124 (53.45) *p* = 0.342	23 (60.53) *p* = 0.251	61 (54.46) *p* = 0.390 *p* = 0.951	84 (56.00) *p* = 0.180 *p* = 0.701
Lead related infective endocarditis probable (with pocket infection or without)	144 (19.05)	38 (16.38) *p* = 0.412	7 (18.42) *p* = 0.908	24 (21.42) *p* = 0.640 *p* = 0.321	31 (20.67) *p* = 0.730 *p* = 0.354
Local (isolated) pocket infection	237 (31.35)	70 (30.17) *p* = 0.797	8 (21.05) *p* = 0.246	27 (24.11) *p* = 0.149 *p* = 0.297	35 (23.33) *p* = 0.063 *p* = 0.188

TLE—transvenous lead extraction, CIED—cardiac implantable electric devices, AAI—pacemaker with one atrial lead, VVI—pacemaker with one ventricular lead, DDD—dual chamber pacemaker, VDD—pacemaker with one ventricular lead, ICD—implantable cardioverter defibrillator, CRTP—cardiac resynchronization therapy pacemaker, CRTD—cardiac resynchronization therapy defibrillator, N—number, sd—standard deviation, IHD—ischemic heart disease, NYHA—New York Heart Association functional class, LVEF—left ventricle ejection fraction.

**Table 3:** System and history of pacing, TLE procedure and potential risk factors for major TLE complications and technical problems.

	Pacemakers All (AAI, VVI, DDD, VDD)	ICDs All (VVI, DDD)	CRT-P	CRT-D	All CRT Systems (CRT-P Plus CRT-D)
	Group 1 N = 756	Group 2 N = 232	Group 3 N = 38	Group 4 N = 112	Group 5 N = 150
	Mean ± sd N (%)	Mean ± sd N (%)	Mean ± sd N (%)	Mean ± sd N (%)	Mean ± sd N (%)
Chi^2^ test,“U” Mann–Whitney test		1 vs. 2	1 vs. 3	1 vs. 42 vs. 4	1 vs. 52 vs. 5
System and history of pacing					
Presence of abandoned lead before TLE	144 (19.05)	20 (8.26) *p* < 0.001	3 (790) *p* = 0.13	21 (18.75) *p* = 0.957 *p* = 0.011	24 (16.00) *p* = 0.446 *p* = 0.041
Number of leads in the heart before TLE	2.04 ± 0.69	1.61 ± 0.65 *p* < 0.001	2.92 ± 0.63 *p* < 0.001	3.13 ± 0.64 *p* < 0.001 *p* < 0.001	3.08 ± 0.64 *p* < 0.001 *p* < 0.001
4 and > 4 in the heart before TLE	34 (4.50)	4 (1.72) *p* = 0.084	4 (10.53) *p* = 0.190	22 (19.64) *p* < 0.001 *p* < 0.001	26 (17.33) *p* < 0.001 *p* < 0.001
Number of procedures before lead extraction	2.21 ± 1.25	1.74 ± 1.00 *p* < 0.001	2.22 ± 1.27 *p* = 0.990	2.39 ± 1.50 *p* = 0.353 *p* < 0.001	2.34 ± 1.44 *p* = 0.407 *p* < 0.001
Time since last CIED procedure (any) (months)	38.72 ± 35.19	29.81 ± 21.99 *p* = 0.021	31.28 ± 25.27 *p* = 0.281	20.70 ± 18.93 *p* < 0.001 *p* = 0.006	23.46 ± 21.39 *p* < 0.001 *p* = 0.022
Potential risk factors for major TLE complications and procedure complexity					
Number of extracted leads in one patient	1.96 ± 0.64	1.57 ± 0.60 *p* < 0.001	2.76 ± 0.71 *p* < 0.001	3.06 ± 0.76 *p* < 0.001 *p* < 0.001	2.99 ± 0.76 *p* < 0.001 *p* < 0.001
Three or more leads were extracted	85 (11.25)	9 (3.88) *p* < 0.001	28 (73.68) *p* < 0.001	96 (85.72) *p* < 0.001 *p* < 0.001	124 (82.67) *p* < 0.001 *p* < 0.001
Utilized approach other than lead venous entry	43 (5.69)	3 (1.24) *p* = 0.009	2 (5.26) *p* = 0.803	2 (1.79) *p* = 0.131 *p* = 0.902	4 (2.67) *p* = 0.186 *p* = 0.557
Extraction of abandoned lead(s) (any)	137 (18.12)	19 (8.19) *p* < 0.001	2 (5.26) *p* = 0.069	21 (18.75) *p* = 0.976 *p* = 0.007	23 (15.33) *p* = 0.483 *p* = 0.044
Oldest extracted lead body dwell time in the patient	106.1 ± 72.73	55.73 ± 45.96 *p* < 0.001	87.16 ± 50.18 *p* = 0.168	61.53 ± 42.43 *p* < 0.001 *p* = 0.177	68.02 ± 45.73 *p* < 0.001 *p* = 0.005
Average extracted lead dwell time in the patient (months)	97.42 ± 63.86	51.45 ± 36.82 *p* < 0.001	68.64 ± 35.96 *p* = 0.007	50.17 ± 31.01 *p* < 0.001 *p* = 0.786	54.85 ± 33.20 *p* < 0.001 0.345
Average lead duration in analyzed group (months)	98.93 ± 69.71	55.26 ± 43.30 *p* < 0.001	68.12 ± 46.87 *p* = 0.018	51.48 ± 38.85 *p* < 0.001 *p* = 0.982	55.43 ± 41.45 *p* < 0.001 *p* = 0.186
Cumulative dwell time of extracted lead (in years) in the patient	16.25 ± 13.13	7.265 ± 6.776 *p* < 0.001	16.13 ± 10.13 *p* = 0.495	13.29 ± 9.472 *p* = 0.053 *p* < 0.001	14.01 ± 9.688 0.190 *p* < 0.001
Number of patients with extracted lead(s) with passive fixation	556 (73.54)	78 (33.62) *p* < 0.001	34 (89.47) *p* = 0.045	106 (94.64) *p* < 0.001 *p* < 0.001	140 (93.33) *p* < 0.001 *p* < 0.001
PADIT score [points]	4.723 ± 2.744	6.135 ± 2.208 *p* < 0.001	4.816 ± 1.625 *p* = 0.269	8.836 ± 1.738 *p* < 0.001 *p* < 0.001	7.581 ± 2.359 *p* < 0.001 *p* < 0.001
SAFeTY-TLE calculator of risk of MC TLE [points]	7.29 ± 4.47	3.95 ± 3.20 *p* < 0.001	7.13 ± 4.34 *p* = 0.786	6.03 ± 3.97 *p* = 0.005 *p* < 0.001	6.31 ± 4.08 *p* = 0.012 *p* < 0.001
SAFeTY-TLE calculator of risk of MC TLE [%]	2 49 ± 4.13	0.88 ± 1.86 *p* < 0.001	2.50 ± 4.87 *p* = 0.868	1.58 ± 2.18 *p* = 0.003 *p* < 0.001	1.82 ± 3.11 *p* = 0.009 *p* < 0.001

TLE—transvenous lead extraction, CIED—cardiac implantable electric devices, AAI—pacemaker with one atrial lead, VVI—pacemaker with one ventricular lead, DDD—dual chamber pacemaker, VDD—pacemaker with one ventricular lead, ICD—implantable cardioverter defibrillator, CRTP—cardiac resynchronization therapy pacemaker, CRTD—cardiac resynchronization therapy defibrillator, MC—major, N—number, sd—standard deviation, MC—major complications.

**Table 4:** TLE complexity in compared groups of patients with different CIED systems.

	Pacemakers All (AAI, VVI, DDD, VDD)	ICDs All (VVI, DDD)	CRT-P	CRT-D	All CRT Systems (CRT-P Plus CRT-D)
	Mean ± sd/N (%)	Mean ± sd/N (%)	Mean ± sd/N (%)	Mean ± sd/N (%)	Mean ± sd/N (%)
Chi^2^ test, “U” Mann–Whitney test		1 vs. 2	1 vs. 3	1 vs. 4 2 vs. 4	1 vs. 5 2 vs. 5
TLE complexity					
Procedure duration (skin to skin) [minutes]	48.45 ± 25.51	42.13 ± 20.94 *p* < 0.001	49.37 ± 17.82 *p* < 0.001	52.65 ± 23.39 *p* < 0.001 *p* < 0.001	51.82 ± 22.10 *p* < 0.001 *p* < 0.001
Procedure duration (sheath to sheath) [minutes]	16.59 ± 24.04	10.79 ± 19.09 *p* < 0.001	17.47 ± 16.90 *p* < 0.001	22.04 ± 23.16 *p* < 0.001 *p* < 0.001	20.88 ± 21.78 *p* < 0.001 *p* < 0.001
Average time of single lead extraction [minutes]	8.11 ± 9.95	6.41 ± 8.93 *p* < 0.001	6.90 ± 7.34 *p* = 0.033	6.99 ± 6.78 *p* = 0.090 *p* = 0.260	6.97 ± 6.90 *p* = 0.013 *p* = 0.358
Technical problems during TLE (any)	144 (19.05)	20 (8.62) *p* < 0.001)	12 (31.58) *p* = 0.094	17 (15.18) *p* = 0.394 *p* = 0.098	29 (19.33) *p* = 0.974 *p* = 0.037
Necessity to change venous approach	53 (7.02)	4 (1.72) *p* = 0.004	2 (5.26) *p* = 0.931	3 (2.68) *p* = 0.125 *p* = 0.857	5 (3.33) *p* = 0.134 *p* = 0.505
Mutual lead to lead connection with strong scar	50 (6.61)	5 (2.16) *p* = 0.015	4 (10.53) *p* = 0.545	9 (8.04) *p* = 0.721 *p* = 0.022	13 (8.67) *p* = 0.467 *p* = 0.007
Break of extracted lead	58 (7.67)	3 (1.29) *p* < 0.001	5 (13.16) *p* = 0.412	3 (2.68) *p* < 0.001 *p* = 0.630	8 (5.33) *p* < 0.001 *p* = 0.046
Byrd dilator collapse/detorsion	20 (2.65)	5 (2.16) *p* = 0.860	1 (2.63) *p* = 0.609	4 (3.57) *p* = 0.803 *p* = 0.681	5 (3.33) *p* = 0.844 *p* = 0.707
Block in venous lead entry region	38 (5.03)	9 (3.88) *p* = 0.588	3 (7.90) *p* = 0.686	9 (8.04) *p* = 0.276 *p* = 0.173	12 (8.00) *p* = 0.207 *p* = 0.135
Two or more technical problems	21 (2.78)	3 (1.29) *p* = 0.298	2 (5.26) *p* = 0.692	5 (4.46) *p* = 0.496 *p* = 0.148	7 (4.67) *p* = 0.336 *p* = 0.044
Utility of additional tools					
Evolution (old and new) or TighRail	6 (0.79)	2 (0.86) *p* = 0.751	2 (5.26) *p* = 0.063	2 (1.79) *p* = 0.620 *p* = 0.832	4 (2.67) *p* = 0.115 *p* = 0.335
Metal sheath	37 (4.89)	9 (3.88) *p* = 0.643	3 (7.89) *p* = 0.656	9 (8.04) *p* = 0.246 *p* = 0.181	12 (8.00) *p* = 0.181 *p* = 0.135
Lasso catheter/snare	25 (3.31)	1 (0.43) *p* = 0.061	1 (2.63) *p* = 0.655	3 (2.68) *p* = 0.811 *p* = 0.852	4 (2.67) *p* = 0.852 *p* = 0.157
Basket catheter	15 (1.98)	1 (0.43) *p* = 0.180	0 (0.00) *p* = 0.790	0 (0.00) *p* = 0.265 *p* = 0.165	0 (0.00) *p* = 0.165 *p* = 0.826
Temporary pacing during procedure	204 (26.99)	19 (8.19) *p* < 0.001	12 (31.58) *p* = 0.664	37 (33.04) *p* = 0.222 *p* = 0.188	49 (32.67) *p* = 0.188 *p* < 0.001

TLE—transvenous lead extraction, CIED—cardiac implantable electric devices, AAI—pacemaker with one atrial lead, VVI—pacemaker with one ventricular lead, DDD—dual chamber pacemaker, VDD—pacemaker with one ventricular lead, ICD—implantable cardioverter defibrillator, CRTP—cardiac resynchronization therapy pacemaker, CRTD—cardiac resynchronization therapy defibrillator, N—number, sd—standard deviation.

**Table 5:** TLE efficacy and complications in compared groups of patients with different CIED systems.

	Pacemakers All (AAI, VVI, DDD, VDD)	ICDs All (VVI, DDD)	CRT-P	CRT-D	All CRT Systems (CRT-P Plus CRT-D)
	Group 1 N = 756	Group 2 N = 232	Group 3 N = 38	Group 4 N = 112	Group 5 N = 150
	N (%)	N (%)	N (%)	N (%)	N (%)
Chi^2^ test,		1 vs. 2	1 vs. 3	1 vs. 4 2 vs. 4	1 vs. 5 2 vs. 5
TLE efficacy and complications					
Major complications (any)	20 (2.65)	1 (0.43) *p* = 0.074	0 (0.00) *p* = 0.628	0 (0.00) *p* = 0.160 *p* = 0.709	0 (0.00) *p* = 0.087 *p* = 0.826
Hemopericardium	10 (1.32)	1 (0.43) *p* = 0.439	0 (0.00) *p* = 0.975	0 (0.00) *p* = 0.453 *p* = 0.709	0 (0.00) *p* = 0.323 *p* = 0.826
Hemothorax	1 (0.13)	0 (0.00) *p* = 0.531	0 (0.00) *p* = 0.823	0 (0.00) *p* = 0.700 MN	0 (0.00) *p* = 0.656 MN
Tricuspid valve damage during TLE (severe)	6 (0.79)	0 (0.00) *p* = 0.380	0 (0.00) *p* = 0.683	0 (0.00) *p* = 0.738 MN	0 (0.00) *p* = 0.587 MN
Rescue cardiac surgery	9 (1.19)	1 (0.43) *p* = 0.525	0 (0.00) *p* = 0.499	0 (0.00) *p* = 0.509 *p* = 0.709	0 (0.00) *p* = 0.372 *p* = 0.826
Death procedure-related (intra-, post-procedural)	1 (0.13)	0 (0.00) *p* = 0.531	0 (0.00) *p* = 0.823	0 (0.00) *p* = 0.700 MN	0 (0.00) *p* = 0.656 MN
Death indication-related (intra-, post-procedural)	4 (0.53)	0 (0.00) *p* = 0.604	0 (0.00) *p* = 0.469	0 (0.00) *p* = 0.981 MN	0 (0.00) *p* = 0.827 MN
Partial radiological success (remaining tip or <4 cm lead fragment)	40 (5.29)	3 (1.29) *p* = 0.015	3 (7.90) *p* = 0.745	1 (0.89) *p* = 0.071 *p* = 0.832	4 (2.67) *p* = 0.247 *p* = 0.557
Full clinical success	710 (93.92)	228 (98.28) *p* = 0.013	34 (89.47) *p* = 0.449	111 (99.11) *p* = 0.041 *p* = 0.902	145 (96.67) *p* = 0.254 *p* = 0.505
Full procedural success	706 (93.39)	228 (98.28) *p* = 0.007	34 (89.47) *p* = 0.545	111 (99.11) *p* = 0.029 *p* = 0.902	145 (96.67) *p* = 0.177 *p* = 0.505

TLE—transvenous lead extraction, CIED—cardiac implantable electric devices, AAI—pacemaker with one atrial lead, VVI—pacemaker with one ventricular lead, DDD—dual chamber pacemaker, VDD—pacemaker with one ventricular lead, ICD—implantable cardioverter defibrillator, CRTP—cardiac resynchronization therapy pacemaker, CRTD—cardiac resynchronization therapy defibrillator, N—number, MN—methodically noncomparable.

**Table 6:** Prognosis in short-, mean- and long-term follow-up in compared groups of patients.

	Pacemakers All (AAI, VVI, DDD, VDD)	ICDs All (VVI, DDD)	CRT-P	CRT-D	All CRT Systems (CRT-P Plus CRT-D)
	Group 1 N = 756	Group 2 N = 232	Group 3 N = 38	Group 4 N = 112	Group 5 N = 150
	N (%)	N (%)	N (%)	N (%)	N (%)
Chi^2^ test,		1 vs. 2	1 vs. 3	1 vs. 42 vs. 4	1 vs. 52 vs. 5
Prognosis in short-, mean- and long-term follow-up					
Alive during 1921 ± 1420 (1–5519) days of follow up	409 (54.10)	110 (47.41) *p* = 0.088	13 (34.21) *p* = 0.026	41 (36.61) *p* < 0.001 *p* = 0.076	54 (36.00) *p* < 0.001 *p* = 0.036
48 h mortality	6 (0.79)	1 (0.43) *p* = 0.380	0 (0.00) *p* = 0.683	0 (0.00) *p* = 0.738 *p* = 0.709	0 (0.00) *p* = 0.587 *p* = 0.826
1 month mortality after TLE; 2–30 days n (% of patients with follow-up longer than 2 days)	19/750 (2.53)	5/231 (2.17) *p* = 0.941	2/38 (5.26) *p* = 0.615	5/112 (4.46) *p* = 0.496 *p* = 0.481	7/150 (4.67) *p* = 0.247 *p* = 0.286
1 year mortality after TLE (31–365 days); n (% of patients with follow-up longer than 30 days)	63/722 (8.73)	31/222 (13.96) *p* = 0.032	8/36 (22.22) *p* = 0.016	24/105 (22.86) *p* < 0.001 *p* = 0.014	32/141 (22.70) *p* < 0.001 *p* = 0.046
3 year mortality after TLE (366–1095 days); n (% of patients with follow-up longer than 365 days)	70/644 (10.87)	33/180 (18.33) *p* = 0.011	8/28 (28.57) *p* = 0.010	24/76 (31.58) *p* < 0.001 *p* = 0.031	32/104 (30.77) *p* < 0.001 *p* = 0.024
Death late > 3 years after TLE (after 1095 days); n (% of patients with follow-up longer than 1095 days)	189/530 (35.66)	52/127 (40.94) *p* = 0.314	7/18 (38.89) *p* = 0.975	18/47 (38.30) *p* = 0.839 *p* = 0.887	25/65 (38.46) *p* = 0.758 *p* = 0.860
All deaths	347 (45.90)	122 (52.59) *p* = 0.880	25 (65.79) *p* = 0.026	71 (63.39) *p* < 0.001 *p* = 0.076	96 (64.00) *p* < 0.001 *p* = 0.036

TLE—transvenous lead extraction, CIED—cardiac implantable electric devices, AAI—pacemaker with one atrial lead, VVI—pacemaker with one ventricular lead, DDD—dual chamber pacemaker, VDD—pacemaker with one ventricular lead, ICD—implantable cardioverter defibrillator, CRTP—cardiac resynchronization therapy pacemaker, CRTD—cardiac resynchronization therapy defibrillator, N—number.

## Data Availability

The data underlying this article will be shared on reasonable request to the corresponding author.
